# Phytochemical and In Silico ADME/Tox Analysis of *Eruca sativa* Extract with Antioxidant, Antibacterial and Anticancer Potential against Caco-2 and HCT-116 Colorectal Carcinoma Cell Lines

**DOI:** 10.3390/molecules27041409

**Published:** 2022-02-19

**Authors:** Amir Mahgoub Awadelkareem, Eyad Al-Shammari, Abd Elmoneim O. Elkhalifa, Mohd Adnan, Arif Jamal Siddiqui, Mejdi Snoussi, Mohammad Idreesh Khan, Z R Azaz Ahmad Azad, Mitesh Patel, Syed Amir Ashraf

**Affiliations:** 1 Department of Clinical Nutrition, College of Applied Medical Sciences, University of Hail, Hail P.O. Box 2440, Saudi Arabia; mahgoubamir22@gmail.com (A.M.A.); eyadhealth@hotmail.com (E.A.-S.); ao.abdalla@uoh.edu.sa (A.E.O.E.); 2 Department of Biology, College of Science, University of Hail, Hail P.O. Box 2440, Saudi Arabia; drmohdadnan@gmail.com (M.A.); arifjamal13@gmail.com (A.J.S.); snmejdi@yahoo.fr (M.S.); 3 Laboratory of Genetic, Biodiversity and Valorization of Bioresources, Higher Institute of Bio-Technology of Monastir, University of Monastir, Avenue Taher Hadded BP 74, Monastir 5000, Tunisia; 4 Department of Clinical Nutrition, College of Applied Health Sciences in Arras, Qassim University, Buraydah 52571, Saudi Arabia; mohdidreesh06@gmail.com or; 5 Department of Post-Harvest Engineering and Technology, Aligarh Muslim University, Aligarh 202002, India; zrazad@gmail.com; 6 Bapalal Vaidya Botanical Research Centre, Department of Biosciences, Veer Narmad South Gujarat University, Surat 395007, India; patelmeet15@gmail.com

**Keywords:** *Eruca sativa*, jarjeer, colorectal cancer, phytochemical analysis, bioactive compounds, erucin, isothiocyanates, nutraceutical

## Abstract

*Eruca sativa* Mill. (*E. sativa*) leaves recently grabbed the attention of scientific communities around the world due to its potent bioactivity. Therefore, the present study investigates the metabolite profiling of the ethanolic crude extract of *E. sativa* leaves using high resolution-liquid chromatography-mass spectrometry (HR-LC/MS), including antibacterial, antioxidant and anticancer potential against human colorectal carcinoma cell lines. In addition, computer-aided analysis was performed for determining the pharmacokinetic properties and toxicity prediction of the identified compounds. Our results show that *E. sativa* contains several bioactive compounds, such as vitamins, fatty acids, alkaloids, flavonoids, terpenoids and phenols. Furthermore, the antibacterial assay of *E. sativa* extract showed inhibitory effects of the tested pathogenic bacterial strains. Moreover, the antioxidant activity of 2,2-diphenyl-1-picrylhydrazyl (DPPH) and hydrogen peroxide (H_2_O_2_) were found to be IC_50_ = 66.16 μg/mL and 76.05 μg/mL, respectively. *E. sativa* also showed promising anticancer activity against both the colorectal cancer cells HCT-116 (IC_50_ = 64.91 μg/mL) and Caco-2 (IC_50_ = 83.98 μg/mL) in a dose/time dependent manner. The phytoconstituents identified showed promising pharmacokinetics properties, representing a valuable source for drug or nutraceutical development. These investigations will lead to the further exploration as well as development of *E. sativa*-based nutraceutical products.

## 1. Introduction

*Eruca sativa* Mill. (*Eruca vesicaria*) is a very popular species of the Brassicaceae family, which is commonly known as arugula, jarjeer or rocket leaves [1,2]. It is also popularly used in salad preparations. *E. sativa* leaves are considered as a valuable source of nutrition due to the presence of several important nutrients, such as dietary fiber, oligosaccharides, amino acids, peptides, proteins, polyunsaturated fatty acids, vitamins, carbohydrates, L-ascorbic acid and mineral content. Furthermore, it has been noticed that *E. sativa* is valued by dieticians for its low calorific content along with high nutritional values [3,4]. 

In recent years, *E. sativa* has come into the limelight due to its rich content of phytochemicals and its significance in various biological activities. Different parts of the *E. sativa* plant possess diverse phytochemicals, such as flavonoids, glucosinolates, phenolics, saponins, tannins and essential oils [5]. Additionally, several phytoconstituents were reported in various studies, namely, isothiocyanates, derivatives of butane, octane, nonane, 4-methylthiobutyl isothiocyanate, cis-3-hexen-1-ol,5-methylthiopentylisothiocyanate, cis-3-hexenyl 2-methylbutanoate, 5-methyl thiopentane nitrile [6], quercetin, kampferol, rutin, myricetin rhamnetin and kaempferol-3-*O*-galactoside [7,8], which indicates that *E sativa* is a rich source of flavonoids that possesses several other important bioactive compounds.

The *E. sativa* seed was investigated for its essential oil content and reported to contain a significant amount of sulfur and nitrogen compounds. Active compounds produced by essential oils, as well as other phytoconstituents during secondary vegetal metabolism are usually considered responsible for biological activities, such as antimicrobial and antioxidant [9]. Additionally, a wide range of phytochemicals present in *E. sativa* were reported to have various biological properties, including antimicrobial, antigenotoxic, antidiuretic, stimulant, stomach disorders analgesic, antioxidant, antiulcer, hepatoprotective activities, antidiabetic, antiacne, antihyperlipidemic, antihyperglycemic and anti-inflammatory [2,10,11,12,13,14]. *E. sativa* seed oil, which is commonly known as taramira oil or jamba oil in Central Asia, is used for massages, hair treatments and as an anti-influenza medication [10]. 

Therefore, based upon its several uses and therapeutic benefits, *E. sativa* grabbed the attention of scientific communities that aimed to further explore, identify and characterize the phytochemicals present in *E. sativa* leaves by using the high resolution-liquid chromatography-mass spectrometry (HR-LC/MS) technique. HR-LC/MS analysis is one of the novel chromatographic techniques usually applied for the identification and quantification of phytochemicals present in plant extracts. HR-LC/MS instrumentation is advantageous for conducting non-targeted analyses, when the compounds of interest are unknown, and it acquires data over a large mass range (e.g., *m*/*z* 100–1500). [15].

An abundance of bioactive components present in *E. sativa* makes it an important leafy vegetable with the potential for nutraceutical purposes and other therapeutic uses. In recent years, a new term, “nutraceuticals”, was introduced by merging two scientific disciplines, “nutrition” and “pharmaceuticals”, which represents any food or part of a food that not only imparts health benefits, but also contributes to the prevention, management or treatment of various diseases [16]. Moreover, in broad terms, nutraceuticals can be summarized as bioactive components, which play a vital role in human beings by maintaining their normal physiological functions and well-being. Hence, *E. sativa* could be further explored for its nutraceutical potential. 

Therefore, based upon the nutraceutical potential and therapeutic benefits, the present study is designed to identify the phytochemicals or bioactive compounds present in *E. sativa* extract using the HR-LC/MS technique. Furthermore, *E. sativa* extract was investigated for several other biological activities, such as antibacterial, antioxidant and anticancer, against the colorectal carcinoma cell lines. Based upon the identification of phytochemicals, computer-aided technology was applied to understand the pharmacokinetic properties as well as toxicity prediction of the identified compounds. According to our report, no study to date has ever reported the potentiality of *E. sativa* crude extract in the management of colorectal cancer with a toxicity prediction of all the phytochemicals identified by HR-LCMS. The obtained results further support in vivo studies for the product’s possible use as a therapeutic, as well as the nutraceutical use of *E. sativa* against various diseases for the management and treatment possibilities of colorectal cancer.

## 2. Results and Discussion

### 2.1. Phytochemical Profiling of E. sativa

**Figure 1:**
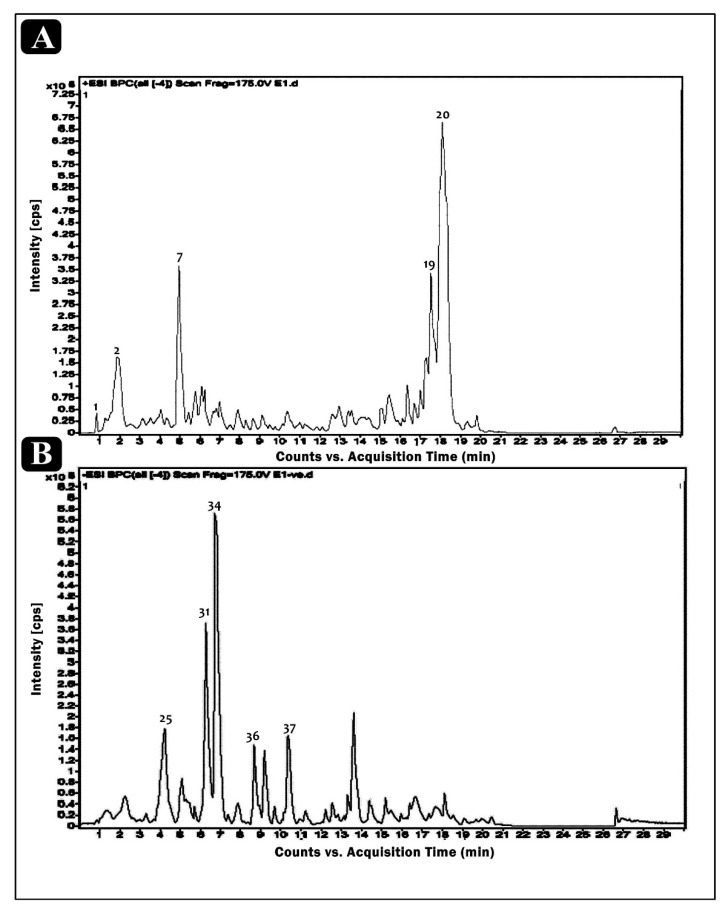
HR-LC/MS spectrum peak of *E. sativa* crude extract showing the chromatogram intensity against the acquisition time, (**A**) positive analysis and (**B**) negative analysis.

**Figure 2:**
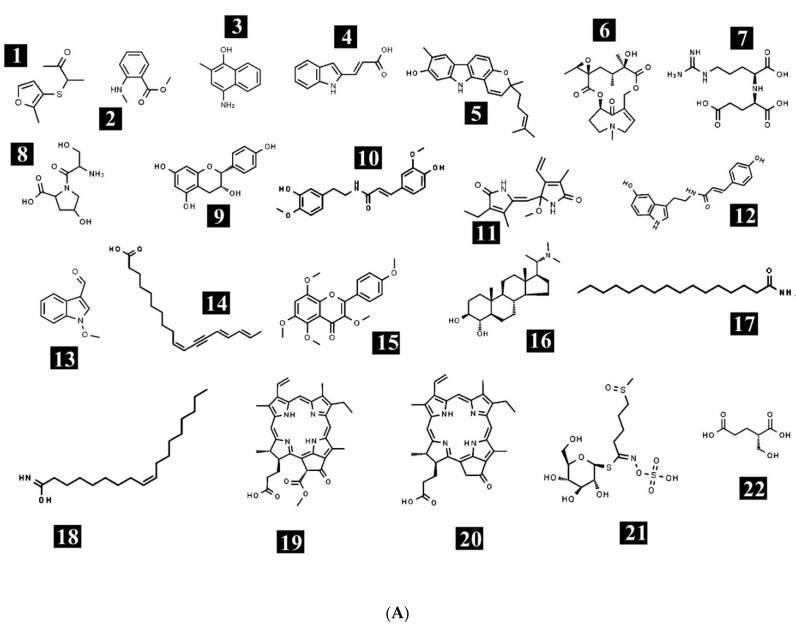

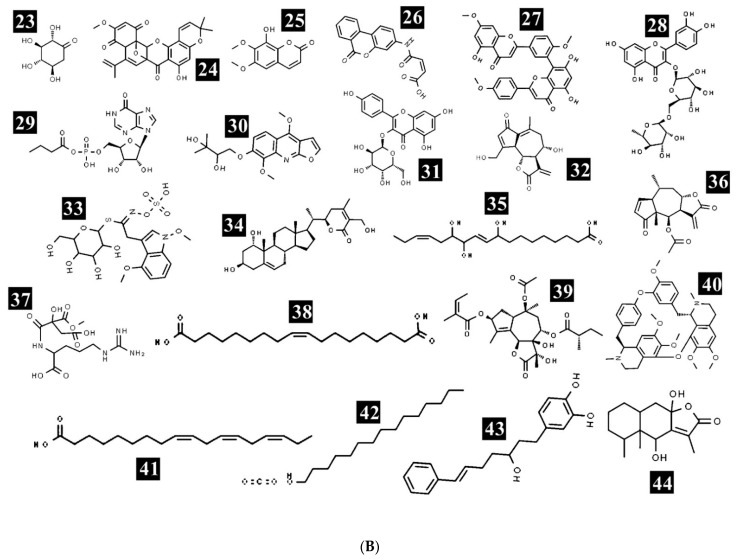
(**A**). Chemical structures of the identified compounds in *E. sativa* crude extract. (1) (+/−)-3-[(2-methyl-3-furyl)thio]-2-butanone; (2) methyl *N*-methylanthranilate; (3) 4-Amino-2-methyl-1-naphthol; (4) indoleacrylic acid; (5) pyrafoline D; (6) petasitenine; (7) nopaline; (8) Serinyl-Hydroxyproline; (9) afzelechin; (10) *N*-trans-Feruloyl-4-*O*-methyldopamine; (11) (±)-rollipyrrole; (12) N6-cis-p-Coumaroylserotonin; (13) 1-Methoxy-1H-indole-3-carboxaldehyde; (14) (10Z,14E,16E)-10,14,16-Octadecatrien-12-ynoic acid; (15) 3,4′,5,6,8-Pentamethoxyflavone; (16) terminaline; (17) palmitic amide; (18) oleamide; (19) pheophorbide a; (20) pyropheophorbide a; (21) glucoraphanin; and (22) (S)-2-(Hydroxymethyl)glutarate. (**B**). Chemical structures of the identified compounds in *E. sativa* crude extract. (23) 2-Deoxy-scyllo-inosose; (24) artomunoxanthentrione epoxide; (25) fraxidin; (26) *N*-(6-Oxo-6H-dibenzo[b,d]pyran-3-yl) maleamic acid; (27) sciadopitysin; (28) rutin; (29) 5′-Butyrylphosphoinosine; (30) evoxine; (31) kaempferol 3-*O*-β-d-galactoside; (32) lactucin; (33) 1,4-Dimethoxyglucobrassicin; (34) pubesenolide; (35) corchorifatty acid F; (36) linifolin A; (37) N2-(2-Carboxymethyl-2-hydroxysuccinoyl)arginine; (38) 9Z-Octadecenedioic acid; (39) trilobolide; (40) thalidasine; (41) α-linolenic acid; (42) 16-Hydroxy hexadecanoic acid; (43) 4-(3-Hydroxy-7-phenyl-6-heptenyl)-1,2-benzenediol; and (44) (6beta,8betaOH)-6,8-Dihydroxy-7(11)-eremophilen-12,8-olide.

The ethanolic crude extract of *E. sativa* was used for tentative phytochemical analysis via HR-LCMS. The% yield of the extract was 4.26%, i.e., 42.60 mg/g of dry weight of the whole plant (*w*/*w*). With the obtained retention times, absorbance spectra and the data of MS, we determined that the chemical composition of the crude extract of *E. sativa* possesses different bioactive compounds (Figure 1). The identified bioactive compounds belong to several classes, such as amino acids, vitamins, fatty acids, alkaloids, flavonoids, terpenoids and phenols. The chemical formula, mass and retention time of the identified compounds are listed in Table 1. The chemical structures of the identified compounds are presented in Figure 2A,B. Some of the bioactive compounds were already identified from different parts of *E. sativa*, such as, 1-methoxy-1H-indole-3-carboxaldehyde from the flower, leaf and seed [17]; 3,4′,5,6,8-pentamethoxyflavone from the fresh leaves [6]; glucoraphanin from the leaves [18]; rutin from the leaves [3,8,18]; kaempferol from the leaves [19] and from the whole plant except the root [7,20,21]; 9Z-Octadecenedioic acid, 16-Hydroxy hexadecanoic acid and (10Z,14E,16E)-10,14,16-Octadecatrien-12-ynoic acid (decanoic acid) from the flower, leaves and seed oil [6,17]; and linolenic acid from the seed oil [1,22,23] and from leaves [19]. In this study, phytoconstituents, namely (+/−)-3-[(2-methyl-3-furyl)thio]-2-butanone; methyl *N*-methylanthranilate; 4-amino-2-methyl-1-naphthol; indoleacrylic acid; pyrafoline D; petasitenine; nopaline; serinyl-Hydroxyproline; afzelechin; *N*-trans-feruloyl-4-*O*-methyldopamine; (±)-rollipyrrole; N6-cis-p-coumaroylserotonin; terminaline; palmitic amide; oleamide; pheophorbide a; pyropheophorbide a; (S)-2-(hydroxymethyl)glutarate; 2-deoxy-scyllo-inosose; artomunoxanthentrione epoxide; fraxidin; *N*-(6-oxo-6H-dibenzo[b,d]pyran-3-yl)maleamic acid; sciadopitysin; 5′-butyrylphosphoinosine; evoxine; lactucin; 1,4-dimethoxyglucobrassicin; pubesenolide; corchorifatty acid F; linifolin A; N2-(2-carboxymethyl-2-hydroxysuccinoyl)arginine; trilobolide; thalidasine; α-linolenic acid; 16-hydroxy hexadecanoic acid; 4-(3-hydroxy-7-phenyl-6-heptenyl)-1,2-benzenediol; and (6beta,8betaOH)-6,8-dihydroxy-7(11)-eremophilen-12,8-olide, were reported, to our knowledge, for the first time to be present in *E. sativa*. The differences in the phytochemistry are varied, possibly due to the season, habitat or the ecological conditions of the plant. 

The presence of different types of phytochemicals, such as flavonoids, phenolics, tannins, saponins and essential oils, are also reported in the literature [1,19,24,25]. The chemodiversity of *E. sativa* essential oils possess 4-methylthiobutyl isothiocyanate, 5-methylthiopentanonitrile, as well as a large amount of sulfur- and nitrogen-containing compounds. Erucic acid is a major component of the plant, along with glucosinolatemethyl sulphinyl butyl isothiocyanate [22]. Furthermore, Jirovetz et al. (2002) analyzed the aromatic compounds of the fresh leaves *E. sativa* using gas chromatography. They reported the presence of various components, such as iso-thiocyanates, derivatives of butane, octane, nonane, 4-methylthiobutyl isothiocyanate, cis-3-hexen-1-ol,5-methylthiopentylisothiocyanate, cis-3-hexenyl 2-methylbutanoate, and 5-methyl thiopentone nitrile [6]. Zhang and Tang (2007) found that *E. sativa* leaves contain volatile components and the major constituents identified were 4-methylthiobutyl isothiocyanate, 5-methylthio-pentanonitrile and abundant amounts of sulfur- and nitrogen-possessing compounds. They also reported that isothiocyanates (isothiocyanate and erucin) had cytoprotective effects [26]. Nazif et al. (2010) investigated a 70% alcoholic extract of *E. sativa* seeds, which led to the isolation of three flavonoids, quercetin, rhamnetin and kaempferol-3-*O*-galactoside, in addition to two glucosinolates; 4-(methylthio)butyl-glucosinolate (glucoerucin) and 3-methylsulfinylpropyl-glucosinolate (glucoiberin) [7]. Michael et al. (2011) determined that the phytochemical analysis of the aqueous extract of *E. sativa* fresh leaves showed nine natural flavonoid compounds. The isolated and identified flavonoids were kaempferol-3-*O*-beta-d-glucoside, rhamnocitrin 3-*O*-(2′′-*O*-methylmalonyl-β-d-glucopyranoside)-4′-*O*-β-d-glucopyranoside, 3-*O*-glucopyranoside, 4′-*O*-glucopyranoside, rhamnocitrin-3-*O*-glucopyranoside, 4′-*O*-glucopyranoside, kaempferol and rhamnocitrin [19].

Arora et al. (2014) revealed that glucosinolates and their hydrolytic products form a significant class of plant secondary metabolites involved in numerous plant defense-linked mechanisms. They exploit the volatile nature of the glucosinolates and develop a method that not only enhance the yield of glucosinolate hydrolytic products, but also reduce the amount of undesired compounds. Among all the tested protocols, the hydro-distillation method using the Clevenger apparatus was considered as the best procedure, which was evident from an enhanced yield as well as an increased number of hydrolytic products when compared to the other methods as observed by gas chromatography-mass spectrometry (GC-MS) [27]. Hussein et al. (2014) revealed that naturally occurring polyphenolic compounds, such as flavonoids, were abundantly present in *E. sativa*, which has a wide range of therapeutic properties (for instance, antimicrobial, anti-allergic, anti-inflammatory, antioxidant and anticancer). Among different classes of flavonoids, the flavonol class was found to be the most important and widely spread flavonoid, in which quercetin, kampferol, rutin and myricetin were found to be the most potent [8]. Cavaiuolo et al. (2014) described *E. sativa* as an important leafy vegetable crop and a good source of antioxidants and anticancer molecules, such as glucosinolates and other sulfur compounds. *E. sativa* is also a hyper-accumulator of nitrates, which have been considered for a long time as the main factors that cause gastro-intestinal cancer. Moreover, recent studies carried out on *E. sativa* determined that the consumption of leafy vegetable would reduce the risk of contracting cancer and other cardiovascular diseases [28].

Bell et al. (2014) concluded that all parts of the *E. sativa* plant, including the seeds, leaves and flowers, contain the chief flavonoids aglycones kaempferol, quercetin and isorhamnetin. The aglycones quercetin and kaempferol are typically found attached to a sugar molecule and acylated. This introduces variations in the biological activities of flavonols. In addition, they reported other major flavonoids, such as kaempferol-di-*O*-glycoside and its isomer, quercetin-di-*O*-glycoside, quercetin-tri-*O*-glycoside, isorhamnetindin-*O*-glycoside, quercetin-3-*O*-glucoside and quercetin-monosinapoyl di-*O*-glycoside, in the *E. sativa* extract [21]. Jalil (2016) estimated four important flavonols (quercetin, kaempferol, rutin and myricetin) and evaluated the antioxidant activity of the methanolic–ethanolic extracts of *E. sativa* leaves. The qualitative and quantitative estimations of quercetin, kaempferol, rutin and myricetin were reported by thin layer chromatography (TLC) and high-performance liquid chromatography (HPLC), revealing that kaempferol had the highest concentration followed by myricetin and rutin, while quercetin had the lowest. It was reported that the antioxidant activity of the *E. sativa* extract was more reactive at a concentration of 20 μg/mL, and less active at a concentration of 2.5 μg/mL (IC_50_ 33 and 47 μg/mL, respectively). Moreover, *E. sativa* has come into the limelight due to its high and significant amount of phytochemicals, which is still under investigation [3].

### 2.2. Antibacterial Activity of E. sativa Crude Extract

**Figure 3:**
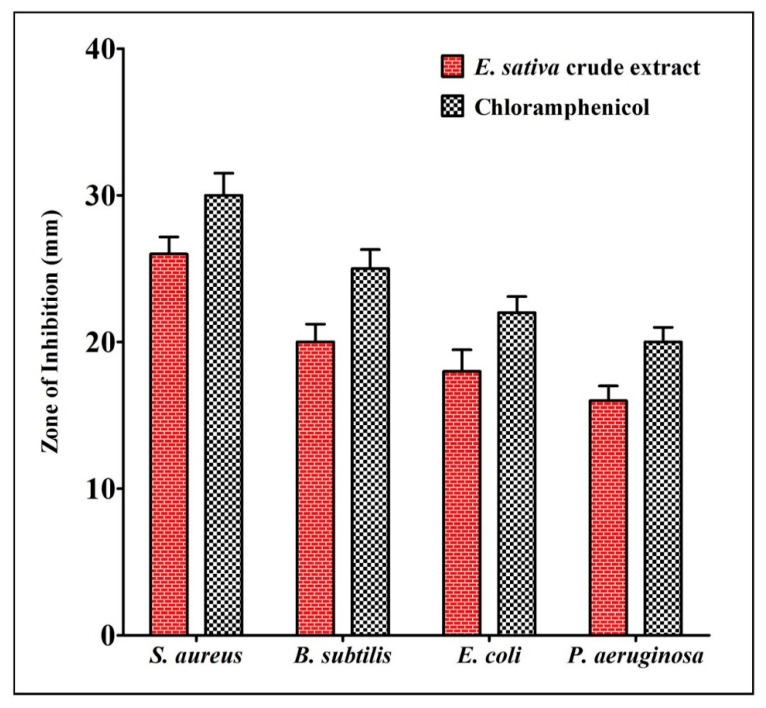
Antibacterial activity (zone of inhibition) against *E. coli*, *P. aeruginosa*, *B. subtilis* and *S. aureus* in comparison with positive control chloramphenicol. The test was carried out in triplicate and the data represent the mean ± SD, *n* = 3.

The antibacterial activity of *E. sativa* crude extract was evaluated via the agar well-diffusion assay method against two strains of Gram-positive pathogenic bacteria (*B. subtilis* and *S. aureus*) and two strains of Gram-negative pathogenic bacteria (*E. coli* and *P. aeruginosa*). Our results show that the crude extract of *E. sativa* exhibits a substantial inhibition of all the tested pathogens. The results of the antibacterial activity as well as the depiction of the crude extract preparation are presented in Figure 3. The obtained results reveal that *E. sativa* crude extract is potentially effective in controlling bacterial growth, and *E. sativa* crude extract shows a maximum zone of inhibition against *S. aureus*, followed by *B. subtilis*, *E. coli* and *P. aeruginosa*.

Currently, the antibiotic resistance of pathogenic bacteria is a major concern around the globe, where multidrug-resistant bacteria are continuously emerging and spreading causing a great challenge to the healthcare system [9]. Therefore, there is always a great demand for the search for an effective novel antimicrobial agent, which can fight against multidrug-resistant bacteria. Plants, especially medicinal/aromatic, are considered as a potential source of antimicrobial agents, as they consist of a high number of diverse phytochemicals [1]. In the present study, the antibacterial activity of *E. sativa* crude extract was evaluated using the agar well-diffusion method against different pathogenic strains of bacteria. Among them, the *E. sativa* crude extract exhibited the considerable inhibition of all the tested pathogens.

Using the agar cup diffusion method, Kauba et al. (2015) reported an inhibition zone (IZ) similar to the one obtained in our results with *E. sativa* ethanolic extract against *S. typhimurium* (IZ = 16.7 mm), *B. subtilis* (IZ = 16.6 mm), *E. coli* (IZ = 16.0 mm) and *B. thuringiensis* (IZ = 15.6 mm) [14]. Additionally, Khoobchandani et al. (2010) reported the antimicrobial activity of crude extracts of different parts of *E. sativa* against two Gram-positive and three Gram-negative bacteria [29]. Among them, a higher activity was reported for the seed oil against Gram-positive bacteria compared to Gram-negative bacteria. Additionally, Qaddoumi and El-banna (2019) reported the antagonistic activity of the aqueous extract of *E. sativa* towards *E. coli* (IZ = 19.0 mm) and *S. aureus* (IZ = 12.0 mm) [30]. In the same study, the antimicrobial activity of the crude extract of ethyl acetate presented no antimicrobial activity towards the tested pathogens. In another study, Rizwan et al. (2016) reported the antimicrobial activities of ethanol, methanol, ethyl acetate, acetone and chloroform extracts from *E. sativa* against different Gram-positive and Gram-negative bacteria [31]. Among them, a higher inhibition activity was found in the ethyl acetate and chloroform extracts against *S. aureus* (IZ = 25.66 mm, 23.16 mm), respectively, followed by methanol and ethanol (IZ = 16 mm, 14.33 mm).

### 2.3. The Antioxidant Activity of E. sativa Crude Extract

Free radicals, such as reactive oxygen species (ROS) and reactive nitrogen species (RNS), are linked with oxygen, which possess a strong reaction activity with other molecules apart from oxygen. Frequently, such free radicals are raised as a by-product of different metabolic activity inside the cell and ionize radiation in the form of the hydroxyl radical (HO•); superoxide ion, O_2_−; singlet oxygen (O_2_); hydrogen peroxide (H_2_O_2_); lipid peroxyl radical (ROO·); lipid alkoxyl radical (RO); lipid hydroperoxide (ROOH); nitrogen di-oxide (·NO_2_); nitric oxide (•NO); peroxynitrite (ONOO-) and thiol radical (RS) [32,33].

These free radicals generate oxidative stress and damage proteins, lipids and nucleic acids. Therefore, the role of free radicals is postulated in different disease conditions, such as the process of aging, various cancers, inflammatory conditions (adult respiratory diseases, arthritis and vasculitis), neurological disorders (Alzheimer’s disease, Parkinson’s disease) and ischemic diseases (stroke, heart diseases and intestinal ischemia) [34].

**Figure 4:**
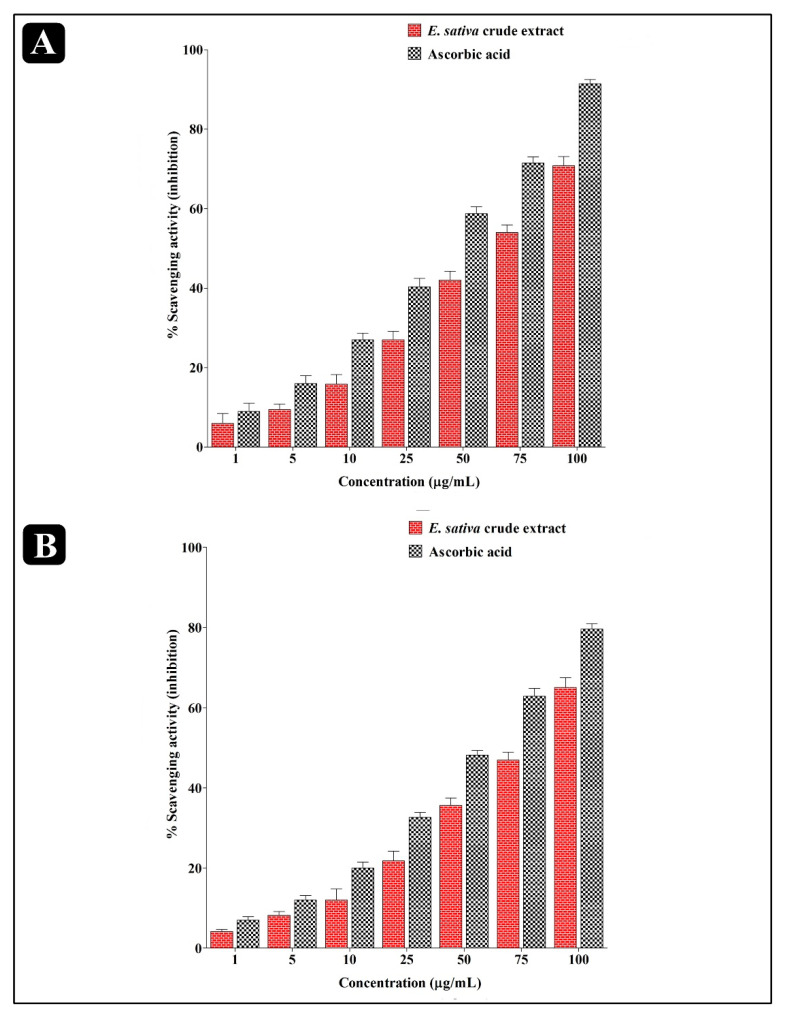
Antioxidant activity of *E. sativa* crude extract against (**A**) DPPH molecules and (**B**) H_2_O_2_ molecules. The activity was carried out in triplicate and the data represent the mean ± SD.

The antioxidant activity of *E. sativa* crude extract was evaluated using very well- established techniques, viz., DPPH and H_2_O_2_ assay. The results of the antioxidant assay reveal that *E. sativa* crude extract can scavenge the radicals to a great extent. The scavenging efficacy of crude extract on DPPH (IC_50_ = 66.16 µg/mL) radicals was better when compared to H_2_O_2_ (IC_50_ = 76.05 µg/mL). Furthermore, the antioxidant potential of *E. sativa* crude extract is dose dependent (Figure 4).

Antioxidants are substances that protect the cells from free radicals. Inside the cell, antioxidant molecules are present at low concentrations, which considerably reduces the oxidative stress and prevents the cells from the damage of free radicals [35]. Apart from endogenous antioxidants, they can also be available exogenously from the diet or as dietary supplements. An efficient antioxidant can readily absorb and remove free radicals and chelate metals at a physiologically appropriate level. Usually, endogenous antioxidants maintain optimal cellular functions, but under the condition of oxidative stress, endogenous antioxidants are not enough and exogenous antioxidants are needed to maintain optimal cellular functions [36]. Plants are considered as the potent source of antioxidant molecules, as they synthesize and accumulate various non-enzymatic antioxidants, such as ascorbic acid, glutathione and phenolics and flavonoids. 

The antioxidant potential of *E. sativa* crude extract was analyzed against DPPH and H_2_O_2_ molecules in comparison to ascorbic acid. The crude extract from *E. sativa* exhibited notable free radical scavenging activity against both DPPH and H_2_O_2_ molecules. Previously, the antioxidant activity of *E. sativa* seeds was tested by Kishore et al. (2016) by using various in vitro antioxidant methods, such as phenolic content, total antioxidant capacity, reducing power, hydrogen peroxide, nitric oxide and superoxide dismutase scavenging activity. According to the previous study, the total phenol content of alcohol extract and hydro-alcohol extract was found to be 216.0 and 229.00 mg/g of gallic acid equivalents, respectively, and the total antioxidant capacity was determined to be 111.00 and 230.60 µM/g of ascorbic acid equivalents. Alcohol extracts and hydro-alcohol extracts were found to be able to scavenge DPPH radicals, hydrogen peroxide radicals, nitric oxide radicals and superoxide radicals, respectively. The IC_50_ values were found to be 3.28 and 3.53 µg/mL, 188.11 and 181.56 µg/mL, 73.05 and 64.33 µg/mL, and 87.91 and 41.12 mg/mL, respectively [37].

Koubaa et al. (2015) reported the antioxidant potential of *E. sativa* leaf extract and concluded that antioxidant activity is present due to the high concentration of phenolics(kaempferol 3,4-di-*O*-glucoside, kaempferol 3-glucosyl, quercetin 3-glucosyl and isorhamnetin 3-glucosyl) [14]. In one more study, Maia et al. (2015) reported the antioxidant potential of the seed extract of *E. sativa* by scavenging H_2_O_2_ and alkyl hydroperoxides accumulated in the cells and peripheral blood via acting as a precursor of sulforaphane, due to the presence of phenolics and glucosinolates in high concentrations in the seed [38].

### 2.4. The Anticancer Activity of E. sativa Crude Extract

**Figure 5:**
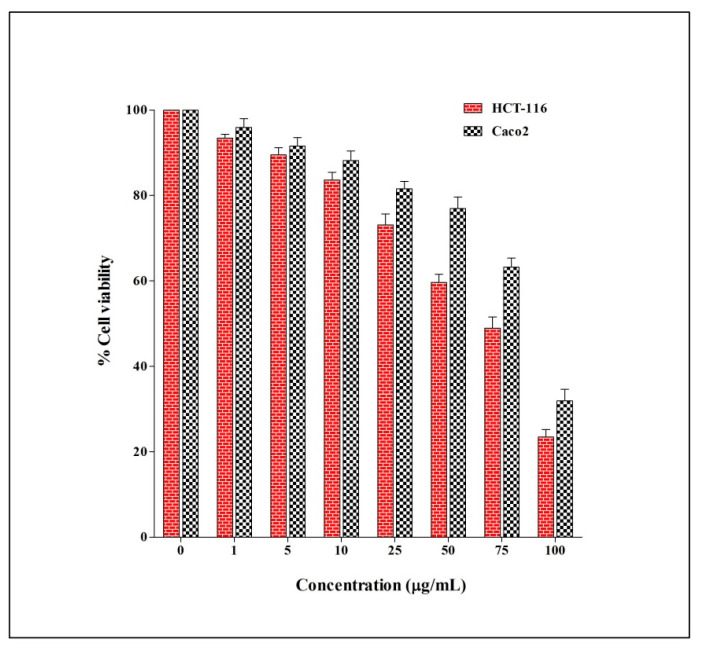
Anticancer activity of *E. sativa* crude extract on human colorectal cancer cell lines (HCT-116 and Caco-2) via MTT assay and the results are expressed in a dose-dependent manner. The activity was carried out in triplicate and the data represent the mean ± SD.

The anticancer potential of *E. sativa* crude extract was evaluated against two different human colorectal cancer cells (HCT-116 and Caco-2) via MTT assay. The results of the anticancer activity reveal that *E. sativa* crude extract could significantly inhibit the cell viability of both cells in a dose-dependent manner. The viability of the HCT-116 cell line after the treatment of crude extract was found to be higher, and the IC_50_ value was calculated, i.e., 64.91 μg/mL compared to Caco-2 with the IC_50_ value of 83.98 μg/mL (Figure 5). 

After cardiovascular diseases, cancer has been reported to be the most common cause of mortality in several countries. In males, lung, liver, colorectal, prostate and stomach cancer are found to be the most common, whereas in females, breast, cervical, lung, thyroid and colorectal cancer are the most common [39]. Therefore, there is an urgent need to find alternate and cost-effective types of cancer treatments with lesser or no side effects. Natural products came to the attention of scientists due to their ability to serve as therapeutic as well as preventive agents over the past decade. Almost 60% of all conventional drugs used in cancer treatment are directly or indirectly extracted from plants, which encourages the discovery of medicinal plant-based novel drugs [39]. There are literatures which states, that the regular consumption of the cruciferous vegetable *E. sativa* can reduce the risk of different types of cancer development in the body. 

Several phytochemicals, such as glucosinolates, sulfur containing plant secondary metabolites and isothiocyanate, including sulforaphane and erucin, are believed to be present in *E. sativa*. Erucin [1-isothiocyanato-4-(methylthio)butane], which is metabolically and structurally similar to sulforaphane, which is also reported to be present in large quantities in *E. sativa* [40]. The promising anticancer effect of erucin was already reported through many in vitro and in vivo studies. In order to observe the protective effect of erucin against cancer, in vivo research was conducted for the first time by a group of researches on the different mouse tissues mediated through the induction of several detoxification enzymes [41]. To date, many different studies confirmed the effectiveness of erucin against several human cancer cells. The anticancer effect of erucin was reported for different human cancer cell lines, such as the lung, liver, colon, and prostate, with the help of different mechanisms, viz., cell cycle regulation, apoptosis and the inhibition of proliferation as well as mitochondrial depolarization. 

One of the studies stated that the erucin can induce apoptosis and also cell cycle arrest on human leukemia cells, together with the multidrug-resistant alternatives [19]. Jakubikova et al. (2005) investigated the anticancer property of erucin, which, similar to sulforaphane, induces the phase II detoxification enzymes and inhibits phase I enzymes. In addition, erucin arrests cell cycle progression and induces apoptosis in human lung carcinomas, hepatomas and leukemia cell lines. Erucin increases the expression of multidrug resistance transporters in human carcinoma cell lines [42]. Nazif et al. (2010) evaluated the cytotoxic activity of the total alcoholic extract of the defatted seeds and the isolated compounds against several types of tumor cell lines using the SRB assay. The total alcoholic extract and the aglucones of the isolated compounds glucoerucin and glucoiberin exhibited significant cytotoxic activity for HCT116 (the colon carcinoma cell line) (IC_50_ = 0.74, 2.42 and 0.94 μg/mL), respectively, while the IC_50_ was >10 μg/mL for Hela (the cervix carcinoma cell line), HEPG2 (the liver carcinoma cell line), MCF7 (the breast carcinoma cell line) and U251 (the brain carcinoma cell line) [7].

Melchini and Maria (2010) revealed that the intake of vegetables is associated with a reduced risk in the development of various types of cancer. This is attributed to the bioactive hydrolysis products that are derived from these vegetables, namely isothiocyanates. Isothiocyanates are characterized as small organic compounds synthesized as glucosinolates with R–N=C=S functional groups. Isothiocyanates present in cruciferous vegetables have a higher amount of anti-cancerous properties and can inhibit cell proliferation. Isothiocyanates suppress cancer cell proliferation by inhibiting the proteins involved in tumor initiation and proliferation pathways. Meanwhile, isothiocyanate treatment stimulates the reactive oxygen species (ROS), cell cycle arrest, programmed cell death and autophagy. More than 20 isothiocyanates are reported as having anticarcinogenic properties against tumorigenesis [43]. Erucin, the isothiocyanate product derived from *E. sativa*, showed chemoprevention activity against cancer cells in animal models. The mechanism of action showed the modulation of phase I, II and III detoxification, the regulation of cell growth by the induction of apoptosis, cell cycle arrest, the induction of ROS mechanisms and regulation androgen receptor pathways [13]. Michael et al. (2011) reported the potent anticancer activity of a 70% ethanolic extract of *E. sativa* against different human tumor cell lines, such as HepG2 (liver carcinoma), MCF7 (breast carcinoma), HCT116 (colon carcinoma) and Hep2 (larynx carcinoma) [19]. Khoobchandani et al. (2011) investigated the anticancer potential of solvent extracts prepared from the aerial roots and seed oil of *E. sativa* against melanoma cells. The seed oil (isothiocyanates rich) was found to significantly reduce the tumor growth and angiogenesis in mice without any major toxicity [19]. Azarenko et al. (2014) found that erucin prevents the proliferation of MCF7 (breast cancer cells) (IC_50_ = 28 mM) in parallel with cell cycle arrest at mitosis (IC_50_ = 13 mM) and apoptosis by a mechanism consistent with the impairment of microtubule dynamics [40]. However, a further exploration using different solvent system extractions and the in vitro anticancer activity of selected cancer cell lines is still required for the therapeutic implications to improve human health.

### 2.5. The Pharmacokinetic and Toxicity (ADMET) Profiles of the Identified Phytoconstituents from E. sativa Ethanolic Crude Extract

The potential of a good drug could be ruined because of the limited absorption, distribution, metabolism, excretion and toxicity (ADMET) characteristics. Furthermore, the major drawback of drug discovery in clinical trials is believed to be its pharmacokinetic properties, by virtue of which it becomes very expensive. Therefore, ADMET parameters were estimated using in silico tools to determine the probability of the *E. sativa* ethanolic crude extract becoming a potential candidate for the development of drugs (Table 2 and Table 3). Interestingly, the majority of the phytoconstituents were found to meet the Lipinski’s rule of five, and some of them were also found to follow Ghose, Veber and Egan filters, with most of them attaining a good score of bioavailability. One more important attribute is the solubility for the absorption of the compound and its distribution in the body, which was specified via the value of aqueous solubility. In the results, it can be observed that most of the compounds are highly soluble in water.

It is important to assess the skin’s permeability, i.e., the rate of a molecule penetrating the stratum corneum, to determine the potential for creating a form of transdermal drug delivery. It is considered that a molecule will penetrate the skin at the log Kp value of more than −2.5 cm/h. All of the identified phytochemical constituents from the crude extract were found to possess moderate-to-good skin penetrability. Caco-2 is the human epithelial colorectal adenocarcinoma cell and its permeability can calculate the intake of oral drugs. Most of the compounds were found to have moderate-to-potent Caco-2 permeability values (log Papp values > 0.90 cm/s).

ADMET analysis is a computer-based drug designing approach, which can lead to the initial stage of drug discovery [44,45,46]. The main motive behind this in silico approach is to lower the cost and time factors involved in comparison to standard ADMET profiling, as the in silico approach can screen more than 20,000 compounds within a minute; the wet lab will take more than 20 weeks to perform the same task [47,48,49]. Therefore, recently, after the establishment of ADMET data in the 1990s, the majority of pharmaceutical companies used this computational approach for the screening of drugs. In the present study, we also performed a computer-aided prediction of pharmacokinetic properties and the safety profile of the identified phytoconstituents via SwissADME and pkCSM tools. The results of the computational analysis can be useful for researchers to advance the development of prospective semi-synthetic and synthetic drugs for miscellaneous use.

Most of the identified phytochemical compounds were not found as a P-gp inhibitor/substrate. P-gp is the main element of ATP-binding cassette transporters or ABC-transporters, which is utilized to protect the central nervous system (CNS) from xenobiotics and is a prime method used to determine active efflux through biological membranes. The majority of the phytochemical compounds were found to be absorbed by the intestine during intestinal absorption analysis. The prediction of skin permeation was carried out by LogKP, by which phytochemical constituents were identified and found to penetrate the skin at a moderate or high level, which confirms their drug-like feature. Compounds 1, 3, 5 and 16 have a log BB (logarithm value of the brain-to-plasma concentration ratio, 0.1 to 0.3) and were determined to have more potential to cross the brain blood barrier (BBB). Only fewer compounds have the ability to penetrate the CNS. The compounds 9, 28, 31 and 43 are amongst the phytoconstituents that are more successfully distributed, with a distribution volume (logVDss) of 0.562 L/kg and 1.444 L/kg, respectively, in the tissues. 

The isoforms of human cytochrome P450 (CYP), which are integrated in the metabolism of drugs inside the liver were also assessed. The foremost clinically appropriate drug-metabolizing enzyme in the human body is CYP3A4. The inhibition of CYP3A4 could direct lead to drug toxicity, drug–drug interactions and other adverse effects. Some of them were non-inhibitors/substrates of isoenzymes. Most of the compounds were found to be non-inhibitors of CYP3A4, the isoenzyme responsible for the metabolism of about 60% of xenobiotics, including drugs, carcinogens, steroids and eicosanoids.

To predict the route of excretion, the total clearance (CLTOT) for both hepatic and renal and renal organic cation transporter 2 (OCT2) substrates was expressed as the log mL/min/kg that was predicted. The results revealed that the majority of the phytochemical constituents displayed a positive total clearance value and can be simply be excreted. The AMES toxicity, hepatotoxicity, hERG potassium channel inhibition and skin sensitization parameters were predicted to find out the toxicity profile of the identified phytochemical constituents from the *E. sativa* crude extract. The obtained results reveal that only a few of the compounds have a deviated mutagenic and hepatic toxicity potential, which means that the majority of the compounds are devoid of any risk of toxicity (99% have no hERG I inhibition and 72% exert no skin-sensitive effects).

**Figure 6:**
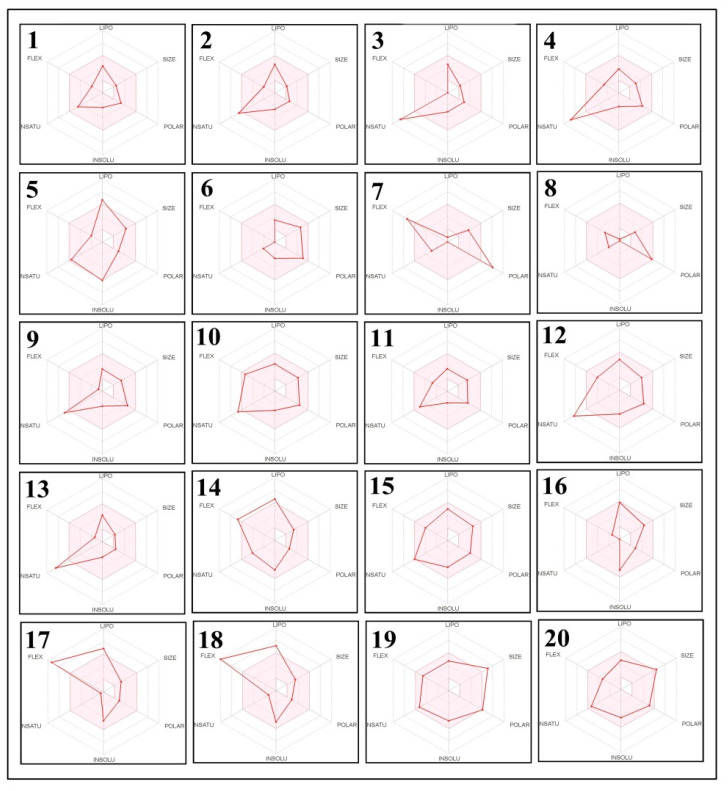
Bioavailability radar of the phytochemical compounds of *E. sativa* based on the physicochemical indices ideal for oral bioavailability. The pink area represents the optimal range for each property (lipophilicity: XLOGP3 between −0.7 and +5.0; size: MW between 150 and 500 g/mol; polarity: TPSA between 20 and 130 Å_2_; solubility: log S not higher than 6; saturation: fraction of carbons in the sp3 hybridization not less than 0.25; and flexibility: no more than 9 rotatable bonds). (1) (+/−)-3-[(2-methyl-3-furyl)thio]-2-butanone; (2) methyl *N*-methylanthranilate; (3) 4-Amino-2-methyl-1-naphthol; (4) indoleacrylic acid; (5) pyrafoline D; (6) petasitenine; (7) nopaline; (8) Serinyl-Hydroxyproline; (9) afzelechin; (10) *N*-trans-Feruloyl-4-*O*-methyldopamine; (11) (±)-rollipyrrole; (12) N6-cis-p-Coumaroylserotonin; (13) 1-Methoxy-1H-indole-3-carboxaldehyde; (14) (10Z,14E,16E)-10,14,16-Octadecatrien-12-ynoic acid; (15) 3,4′,5,6,8-Pentamethoxyflavone; (16) terminaline; (17) palmitic amide; (18) oleamide; (19) pheophorbide; and (20) pyropheophorbide a.

**Figure 7:**
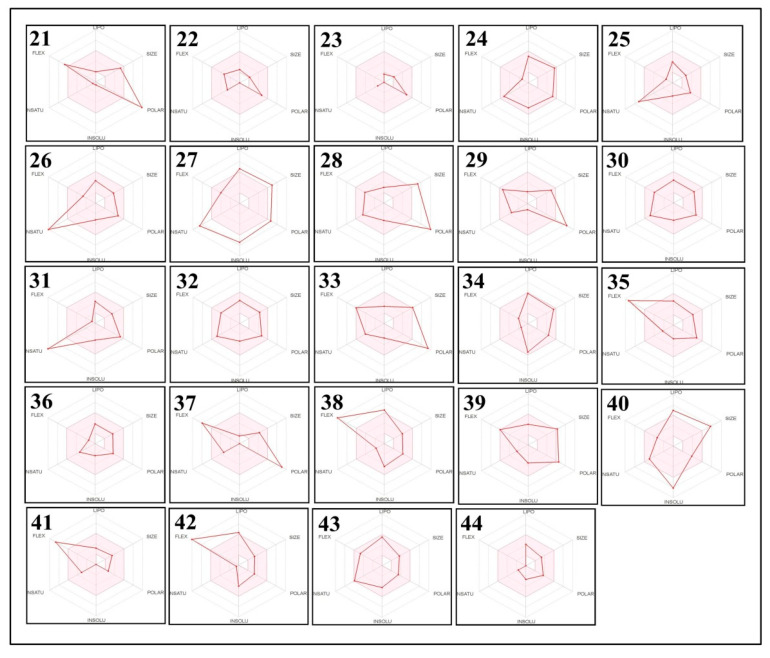
Bioavailability radar of the phytochemical compounds of *E. sativa* based on the physicochemical indices ideal for oral bioavailability. The pink area represents the optimal range for each property (lipophilicity: XLOGP3 between −0.7 and +5.0; size: MW between 150 and 500 g/mol; polarity: TPSA between 20 and 130 Å_2_; solubility: log S not higher than 6; saturation: fraction of carbons in the sp3 hybridization not less than 0.25; and flexibility: no more than 9 rotatable bonds). (21) Glucoraphanin; (22) (S)-2-(Hydroxymethyl)glutarate;, (23) 2-Deoxy-scyllo-inosose; (24) artomunoxanthentrione epoxide; (25) fraxidin; (26) *N*-(6-Oxo-6H-dibenzo[b,d]pyran-3-yl) maleamic acid; (27) sciadopitysin; (28) rutin; (29) 5′-Butyrylphosphoinosine; (30) evoxine; (31) kaempferol 3-*O*-β-d-galactoside; (32) lactucin; (33) 1,4-Dimethoxyglucobrassicin; (34) pubesenolide; (35) corchorifatty acid F; (36) linifolin A; (37) N2-(2-Carboxymethyl-2-hydroxysuccinoyl)arginine; (38) 9Z-Octadecenedioic acid; (39) trilobolide; (40) thalidasine; (41) α-linolenic acid; (42) 16-Hydroxy hexadecanoic acid; (43) 4-(3-Hydroxy-7-phenyl-6-heptenyl)-1,2-benzenediol; and (44) (6beta,8betaOH)-6,8-Dihydroxy-7(11)-eremophilen-12,8-olide.

To obtain further information about the improved bioavailability and drug-likeness of the identified phytochemicals, we estimated the results of the bioavailability radar. The results are represented by the lipophilicity: XLOGP3 between −0.7 to +5.0; polarity: TPSA between 20–130 Å_2_; size: molecular weight 150–500 g/mol; saturation: fraction of carbons in the sp3 hybridization not less than 0.25; solubility: log S not higher than 6; and flexibility: no more than 9 rotatable bonds with the colored zone representing the desired physico-chemical space for good oral bioavailability, and they all display significant drug-likeness properties (Figure 6 and Figure 7).

**Figure 8:**
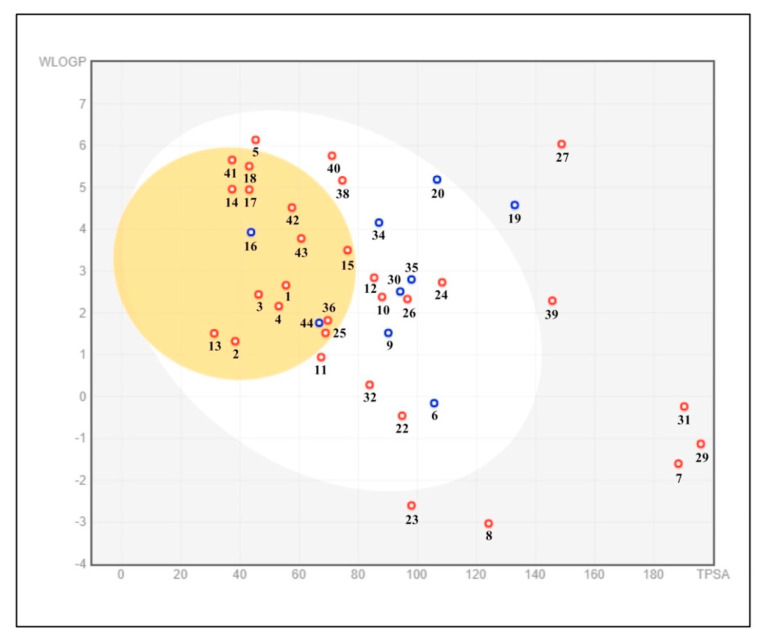
BOILED Egg model of the phytochemical compounds of *E. sativa* using the Swiss ADME predictor. BOILED Egg allows for the intuitive evaluation of passive gastrointestinal absorption (HIA) and brain penetration (BBB) in function of the position of the molecules in the WLOGP-versus-TPSA referential. The white region indicates the high probability of passive absorption by the gastrointestinal tract, and the yellow region (yolk) indicates the high probability of brain penetration. The yolk and white areas are not mutually exclusive. In addition, the points are colored in blue if predicted as actively effluxed by P-gp (PGP+) and in red if predicted as non-substrate of P-gp (PGP-).

The examples of the brain or intestinal estimated permeation (BOILED Egg model) estimation of gastrointestinal (GI) absorption and BBB permeation for all the identified compounds were conducted. The results show that the compound indicated with a red point in the yellow ellipse has the potential for brain penetration and is a non-substrate of P-gp (PGP-) (Figure 8).

Some of the plant families, such as Brassicaceae, Capparaceae and Resedaceae, were reported to contain glucosinolates, which are considered as one of the most significant secondary metabolites. Subsequently, in addition to the enzyme myrosinase, secondary metabolites are broken down into various hydrolytic products [50,51]. *E. sativa*, a member of the Brassicaceae family, is reported to be a rich source of glucosinolates, and its different hydrolytic products are reported to exert different bioactive properties, such as anticancer, antimutagenic, bioherbicidal, antimicrobial, antigenotoxic and antitumor activities [52,53,54,55,56]. In the present study, two glucosinolates, glucoraphanin and 4-dimethoxyglucobrassicin, were identified, which could be the major constituents of the *E. sativa* crude extract and are possibly responsible for various potent biological activities.

## 3. Materials and Methods

### 3.1. Media and Chemicals

All the microbiological media used in the present study were purchased from the local suppliers of Hi-Media^®^, Mumbai, India. Chemicals, drugs and solvents were purchased from Sigma-Aldrich^®^, Bangalore, India. Analytical grade solvents were used.

### 3.2. Plant Material and Extraction Preparation

*E. sativa* plants were cultivated in a field for the purpose of the present study. Firstly, the plants of *E. sativa* were grown in silty clay with sand soil type at an average temperature of 30–35 °C, and were watered once every day. Later, the whole plant leaves were collected after maturity, washed under running tap water and oven-dried. Dry plant leaves were then grounded into a fine powder with an electrical grinder and stored in airtight containers. Next, *E. sativa* powder (25 g) was soaked in 85% ethanol at 37 °C for 24 h with vigorous shaking at 120 rpm. The ethanolic extract was filtered using Whatman no. 1 filter paper and the obtained extract was concentrated using a rotary evaporator. The obtained dried residues were dissolved in 10% dimethyl sulfoxide (DMSO) to make up 1 mg/mL of plant leaf extract concentration to carry out various biological assays [57].

### 3.3. High Resolution Liquid Chromatograph Mass Spectrometery Analysis

The HR-LCMS analysis of the ethanolic extract was carried out by a UHPLC-PDA-Detector Mass Spectrophotometer (HR-LC/MS 1290 Infinity UHPLC System), Agilent Technologies^®^, Santa Clara, CA, USA, and consisted of an HiP sampler, binary gradient solvent pump, column compartment and Quadrupole Time of Flight Mass Spectrometer (MS Q-TOF) with a dual Agilent Jet Stream Electrospray (AJS ES) ion source. A total of 1% formic acid in deionized water (solvent A) and acetonitrile (solvent B) was used as a solvent. The flow rate of 0.350 mL/min was used, while MS detection was performed in MS Q-TOF. Compounds were identified via their mass spectra and their unique mass fragmentation patterns. Compound Discoverer 2.1, ChemSpider and PubChem were used as the main tools for the identification of the phytochemical constituents [58].

### 3.4. Antibacterial Assay

All pathogenic bacterial test strains, *Pseudomonas aeruginosa* (*P. aeruginosa*) (MTCC 741), *Bacillus subtilis* (*B. subtilis*) (MTCC 121), *Escherichia coli*, (*E. coli*) (MTCC 9537) and *Staphylococcus aureus* (*S. aureus*) (MTCC 96), were obtained from the Microbial Type Culture Collection (MTCC), India, and Muller–Hinton Agar (MHA) was used to maintain the bacterial culture. The antibacterial activity of *E. sativa* crude extract was carried out via the agar cup/well diffusion method. Firstly, bacterial cultures were grown overnight at 37 °C in fresh medium and a total of 0.5 McFarland standard 10^8^ colony-forming units/mL (CFU/mL) was matched by culture turbidity adjustment using 0.9% of sterile saline solution. Bacterial suspension was evenly spread all over the plates, and the wells were made with a sterile cork borer. A total of 60 µL of crude extract (1 mg/mL) was then inoculated into each respective well and the plates were incubated at 37 °C for 24 h. Antibacterial activity was noted in the form of the zone of inhibition. A total of 1000 µg/mL of chloramphenicol and 10% of DMSO was used as a positive and negative control, respectively [58]. 

### 3.5. Antioxidant Assays

#### 3.5.1. DPPH Scavenging Activity

The antioxidant potential of *E. sativa* crude extract was determined in terms of its radical scavenging capability against DPPH free radicals [59]. Crude extracts of different concentrations (1–100 µg/mL) were mixed in the tubes containing 2 mL of DPPH solution (6 × 10^−5^ M) in DMSO. The tubes were mixed well and incubated in the dark for 1 h. At the end of the incubation, the absorbance was read out at 517 nm. DMSO was used as a blank, DPPH solution without any crude extract was used as a control, and ascorbic acid was used as a standard [60]. The calculation of the percentage of scavenging of DPPH free radicals was calculated as follows:

DPPH scavenging activity (%) = (A0 − A1)/A0 × 100(1)

where A0 = absorbance of the control; A1 = absorbance of the sample.

#### 3.5.2. Hydrogen Peroxide Scavenging Activity

The antioxidant potential of *E. sativa* crude extract against H_2_O_2_ was carried out according to the described method [59]. Crude extract of different concentrations (1–100 µg/mL) were mixed in the tubes having 1 mL of H_2_O_2_ (1 mL, 2 mM) solution, prepared in a phosphate buffer (0.1M, pH 7.4). The tubes were then incubated for 10 min at room temperature. At the end of the incubation, the absorbance was read out at 230 nm against a blank solution (phosphate buffer without H_2_O_2_), while ascorbic acid was used as a positive control [61]. The following formula was then used to calculate the percentage of H_2_O_2_ scavenged:

Inhibition (%) = (A0 − A1)/A0) × 100(2)

where A0 = absorbance of the control; A1 = absorbance of the extract/standard

### 3.6. Anticancer Assay (MTT Assay)

The anticancer activity of *E. sativa* crude extract was carried out against human colon cancer cell lines (HCT-116 and Caco-2). Both cells were acquired from the National Centre for Cell Science (NCCS), India, and propagated in 25 cm^2^ flask containing Dulbecco’s Modified Eagle’s Medium (DMEM) supplemented with 10% Fetal Bovine Serum (FBS) and antibiotic solution in a humidified (5% CO_2_) atmosphere at 37 °C. To perform the MTT assay, cells were grown up to 80% confluence and then seeded at a density of more than 1 × 105 cells per well in 96-well plates and incubated in conditions, as mentioned above. Trypan Blue (0.4%) was used to stain the cells, and the viability was calculated by using a hemocytometer. Cells were then treated with different concentrations of *E. sativa* crude extract (1–100 μg/mL) for 24 h. Cells were washed with PBS solution and subjected to 100 μL of MTT solution (3-(4,5-dimethylthiazolyl-2)-2,5 diphenyltetrazolium bromide) (5 mg/mL) and incubated for 4 h. Then, the medium was removed and 100 μL of DMSO was added to solubilize the formazan crystals. The amount of formazan crystal was determined by measuring the absorbance at 570 nm using an ELISA reader. Cisplatin was used as a positive control. All assays were performed in triplicate and 50% cytotoxic concentration (IC_50_) was calculated [62,63].

### 3.7. ADMET Analysis

Assumption of the toxicity and pharmacokinetics of the compounds identified from *E. sativa* crude extract via HR-LC/MS was carried out via SwissADME (http://www.swissadme.ch/) and pkCSM (http://biosig.unimelb.edu.au/pkcsm/prediction) online tools accessed on 7 September 2021 [64,65,66].

## 4. Conclusions

From the present study, it can be concluded that *E. sativa* displays potent antibacterial activity against the different human pathogenic bacterial strains. Additionally, *E. sativa* also shows promising antioxidant and anticancer activity. Based on our results, *E. sativa* can also be utilized for the production and development of nutraceutical or functional food. Therefore, further investigation warrants in vivo pharmacological and toxicological research to demonstrate the unexplored and valuable aspect of *E. sativa*. The computational analysis shows that *E. sativa* possesses a significant pharmacokinetic and safety profile of the phytochemical constituents identified using HR-LC/MS. Additionally, it also reveals the ethanolic crude extract of *E. sativa* as a prospective drug candidate for the treatment and management of various diseases, as well as for therapeutic purposes. However, the current computational models are severely constrained due to the lack of understanding of the underlying molecular processes of the diseases. Therefore, to overcome these limitations, one way to formulate more effective strategies is to incorporate ligand, target, phenotype and biological network-based approaches, as well as a deeper reinforcement of learning methods, which are more likely to increase predictability. This will overcome the shortcomings of the existing computational approaches and improve the drug development process. 

## Figures and Tables

**Table 1:** Identified tentative phytoconstituents from the ethanolic crude extract of *E. sativa* using HR-LC/MS.

Compound Number	Analysis Mode	Name	Class	Formula	Mass	*m*/*z*	RT
1	Positive	(+/−)-3-[(2-methyl-3-furyl)thio]-2-butanone	Furans-aryl thioethers	C_9_H_12_O_2_S	184.0576	185.0649	0.816
2	Positive	Methyl *N*-methylanthranilate	Methyl ester	C_9_H_11_NO_2_	165.078	166.0852	2.06
3	Positive	4-Amino-2-methyl-1-naphthol	Vitamin	C_11_H_11_NO	173.0859	174.0937	2.55
4	Positive	Indoleacrylic acid	Indoles	C_11_H_9_NO_2_	187.0623	188.0696	3.189
5	Positive	Pyrafoline D	Carbazoles	C_23_H_25_NO_2_	347.1928	348.2001	3.705
6	Positive	Petasitenine	Spiro-epoxide	C_19_H_27_NO_7_	381.1773	382.1846	4.232
7	Positive	Nopaline	Amino acid	C_11_H_20_N_4_O_6_	304.1444	305.152	5.05
8	Positive	Serinyl-Hydroxyproline	Dipeptide	C_8_H_14_N_2_O_5_	218.0894	219.0974	5.443
9	Positive	Afzelechin	Flavonoid	C_15_H_14_O_5_	274.079	275.0873	6.004
10	Positive	*N*-trans-Feruloyl-4-*O*-methyldopamine	Cinnamamides	C_19_H_21_NO_5_	343.1368	344.145	6.22
11	Positive	(±)-Rollipyrrole	Pyrrolines	C_16_H_20_N_2_O_3_	288.1463	289.1536	6.853
12	Positive	N6-cis-p-Coumaroylserotonin	*N*-acylserotonins	C_19_H_18_N_2_O_3_	322.1306	323.1379	7.024
13	Positive	1-Methoxy-1H-indole-3-carboxaldehyde	Indoles	C_10_H_9_NO_2_	175.0625	176.0697	7.09
14	Positive	(10Z,14E,16E)-10,14,16-Octadecatrien-12-ynoic acid	Fatty acids	C_18_H_26_O_2_	274.192	275.1991	8.672
15	Positive	3,4′,5,6,8-Pentamethoxyflavone	Flavonoids	C_20_H_20_O_7_	372.1191	373.1264	11.085
16	Positive	Terminaline	Corticosteroid	C_23_H_41_NO_2_	363.3122	364.3195	12.545
17	Positive	Palmitic amide	Fatty amide	C_16_H_33_NO	255.2554	256.2627	16.876
18	Positive	Oleamide	Fatty amide	C_18_H_35_NO	281.2705	282.2778	17.242
19	Positive	Pheophorbide a	-	C_35_H_36_N4O_5_	592.2665	593.2738	17.474
20	Positive	Pyropheophorbide a	-	C_33_H_34_N_4_O_3_	534.2615	535.2688	17.918
21	Negative	Glucoraphanin	Thia-glucosinolic acid	C_12_H_23_NO_10_S_3_	437.0439	436.0367	1.124
22	Negative	(S)-2-(Hydroxymethyl)glutarate	-	C_6_H_10_O_5_	162.0501	161.0429	1.202
23	Negative	2-Deoxy-scyllo-inosose	Cyclohexanone	C_6_H_10_O_5_	162.0502	161.0431	1.485
24	Negative	Artomunoxanthentrione epoxide	Pyranoxanthones	C_26_H_22_O_8_	462.1337	461.1265	3.084
25	Negative	Fraxidin	Hydroxycoumarins	C_11_H_10_O_5_	222.0532	221.0463	3.883
26	Negative	*N*-(6-Oxo-6H-dibenzo[b,d]pyran-3-yl)maleamic acid	Coumarins	C_17_H_11_NO_5_	309.067	354.0659	4.262
27	Negative	Sciadopitysin	Flavonoid	C_33_H_24_O_10_	580.1389	625.1373	5.015
28	Negative	Rutin	Flavonoid	C_27_H_30_O_16_	610.1504	609.1432	5.015
29	Negative	5′-Butyrylphosphoinosine	-	C_14_H_19_N_4_O_9_P	418.0875	463.0856	5.735
30	Negative	Evoxine	Alkaloid	C_18_H_21_NO_6_	347.1374	392.136	6.06
31	Negative	Kaempferol 3-*O*-β-d-galactoside	-	C_21_H_20_O_11_	448.0983	447.0911	6.223
32	Negative	Lactucin	Gamma butyrolactones	C_15_H_16_O_5_	276.1009	321.0994	6.227
33	Negative	1,4-Dimethoxyglucobrassicin	Indole glucosinolate	C_18_H_24_N_2_O_11_S_2_	508.0794	507.0722	6.294
34	Negative	Pubesenolide	-	C_28_H_42_O_5_	458.2963	457.2891	7.746
35	Negative	Corchorifatty acid F	Fatty acid	C_18_H_32_O_5_	328.2228	327.2156	8.681
36	Negative	Linifolin A	Terpenoid	C_17_H_20_O_5_	304.1319	349.1304	8.903
37	Negative	N2-(2-Carboxymethyl-2-hydroxysuccinoyl)arginine	Amino acid	C_12_H_20_N_4_O_8_	348.1297	393.1286	10.612
38	Negative	9Z-Octadecenedioic acid	Fatty acid	C_18_H_32_O_4_	312.2278	311.2204	11.488
39	Negative	Trilobolide	Terpenoid	C_27_H_38_O_10_	522.2548	521.2495	12.359
40	Negative	Thalidasine	Alkaloid	C_39_H_44_N_2_O_7_	652.3179	711.3339	12.591
41	Negative	α-linolenic acid	Fatty acid	C_18_H_30_O_2_	278.2223	277.2151	16.479
42	Negative	16-Hydroxy hexadecanoic acid	Fatty acid	C_16_H_32_O_3_	272.2328	271.2256	16.487
43	Negative	4-(3-Hydroxy-7-phenyl-6-heptenyl)-1,2-benzenediol	-	C_19_H_22_O_3_	298.1575	297.1505	17.381
44	Negative	(6beta,8betaOH)-6,8-Dihydroxy-7(11)-eremophilen-12,8-olide	Terpenoids	C_15_H_22_O_4_	266.1527	265.1458	17.524

**Table 2:** Absorption, distribution, metabolism, excretion and toxicity (ADMET) properties of the identified phytochemical compounds of *E. sativa*: (1) (+/-)-3-[(2-methyl-3-furyl)thio]-2-butanone; (2) methyl *N*-methylanthranilate; (3) 4-Amino-2-methyl-1-naphthol; (4) indoleacrylic acid; (5) pyrafoline D; (6) petasitenine; (7) nopaline; (8) serinyl-hydroxyproline; (9) afzelechin; (10) *N*-trans-Feruloyl-4-*O*-methyldopamine; (11) (±)-rollipyrrole; (12) N6-cis-p-Coumaroylserotonin; (13) 1-Methoxy-1H-indole-3-carboxaldehyde; (14) (10Z,14E,16E)-10,14,16-Octadecatrien-12-ynoic acid; (15) 3,4′,5,6,8-Pentamethoxyflavone; (16) terminaline; (17) palmitic amide; (18) oleamide; (19) pheophorbide a; and (20) pyropheophorbide a.

Entry	01	02	03	04	05	06	07	08	09	10	11	12	13	14	15	16	17	18	19	20
**Drug-Likeness**																				
**Lipinski**	Yes	Yes	Yes	Yes	Yes	Yes	Yes	Yes	Yes	Yes	Yes	Yes	Yes	Yes	Yes	Yes	Yes	Yes	Yes	Yes
**Bioavailability score**	0.55	0.55	0.55	0.56	0.55	0.55	0.11	0.55	0.55	0.55	0.55	0.55	0.55	0.85	0.55	0.55	0.55	0.55	0.56	0.56
**Absorption**																				
**Water** **solubility**	−2.128	−1.264	−2.845	−3.419	−5.033	−3.47	−2.892	−2.236	−3.254	−3.438	−3.044	−3.73	−1.522	−5.475	−4.782	−3.24	−6.511	−7.074	−4.432	−4.517
**Caco2** **permeability**	1.638	1.747	1.183	0.903	1.159	0.639	−0.55	−0.382	1.077	1.031	0.684	0.812	1.86	1.597	1.245	1.142	1.525	1.55	0.538	0.603
**Intestinal** **absorption** **(human)**	95.166	93.334	91.703	91.239	90.172	83.228	0.00	29.521	91.482	90.302	94.956	90.379	97.487	94.648	98.581	88.19	90.399	90.218	70.994	83.4
**Skin** **Permeability**	−2.164	−2.165	−2.743	−2.717	−2.752	−2.777	−2.735	−2.735	−2.735	−2.786	−3.917	−2.738	−2.107	−2.717	−2.672	−3.106	−2.565	−2.725	−2.735	−2.734
**P-glycoprotein substrate**	No	No	Yes	Yes	Yes	Yes	Yes	Yes	Yes	Yes	No	Yes	Yes	No	No	Yes	No	No	No	No
**P-glycoprotein I inhibitor**	No	No	No	No	No	No	No	No	No	No	No	No	No	No	Yes	Yes	No	No	No	No
**P-glycoprotein II inhibitor**	No	No	No	No	Yes	No	No	No	No	No	No	No	No	No	Yes	No	No	No	Yes	Yes
**Distribution**																				
**VDss (human)**	0.0006	−0.15	0.322	−0.905	0.19	0.353	0.007	−0.842	0.562	0.053	−0.067	0.053	0.088	−0.702	−0.228	−0.266	0.319	0.281	−0.678	−0.434
**BBB** **permeability**	0.22	−0.087	0.333	−0.746	0.163	−0.701	−1.329	−0.793	−0.818	−0.834	−0.567	−0.808	0.094	−0.036	−1.026	0.182	−0.332	−0.389	−0.888	−0.722
**CNS** **permeability**	−2.759	−1.785	−1.873	−2.411	−1.361	−3.053	−4.282	−4.104	−2.473	−2.682	−2.992	−2.326	−2.126	−1.387	−3.022	−2.298	−1.813	−1.651	−2.116	−1.779
**Metabolism**																				
**CYP2D6** **substrate**	No	No	No	No	No	No	No	No	No	No	No	Yes	No	No	No	No	No	No	No	No
**CYP3A4** **substrate**	No	No	Yes	No	Yes	No	No	No	No	Yes	No	Yes	No	Yes	Yes	Yes	Yes	Yes	Yes	Yes
**CYP1A2** **inhibitor**	No	No	Yes	No	Yes	No	No	No	No	No	No	Yes	Yes	Yes	Yes	No	Yes	Yes	No	No
**CYP2C19** **inhibitor**	No	No	No	No	Yes	No	No	No	No	No	No	Yes	No	No	Yes	No	No	No	No	No
**CYP2C9** **inhibitor**	No	No	No	No	Yes	No	No	No	No	No	No	Yes	No	No	Yes	No	No	No	No	No
**CYP2D6** **inhibitor**	No	No	No	No	No	No	No	No	No	No	No	No	No	No	No	Yes	No	No	No	No
**CYP3A4** **inhibitor**	No	No	No	No	Yes	No	No	No	No	No	No	Yes	No	Yes	Yes	No	No	No	No	No
**Excretion**																				
**Total clearance**	0.371	0.75	0.302	0.644	0.343	0.627	−0.171	0.339	0.255	0.271	0.583	0.454	0.297	1.917	0.769	0.206	1.837	1.959	0.135	0.213
**Renal OCT2 substrate**	No	No	No	No	No	No	No	No	No	No	No	No	No	No	No	No	No	No	No	No
**Toxicity (Compound Number)**																				
**AMES toxicity**	No	No	Yes	No	Yes	Yes	No	No	No	No	No	No	No	No	No	No	No	No	No	No
**Hepatotoxicity**	No	No	No	No	Yes	Yes	No	No	No	No	No	Yes	No	Yes	No	No	No	No	Yes	Yes
**hERG I inhibitors**	No	No	No	No	No	No	No	No	No	Yes	No	No	No	No	No	No	No	No	No	No
**Skin Sensitization**	Yes	Yes	Yes	No	Yes	No	No	No	No	No	No	No	Yes	Yes	No	No	Yes	Yes	No	No

**Table 3:** Absorption, distribution, metabolism, excretion and toxicity (ADMET) properties of the identified phytochemical compounds of *E. sativa*: (21) glucoraphanin; (22) (S)-2-(Hydroxymethyl)glutarate; (23) 2-Deoxy-scyllo-inosose; (24) artomunoxanthentrione epoxide; (25) fraxidin; (26) *N*-(6-Oxo-6H-dibenzo[b,d]pyran-3-yl) maleamic acid; (27) sciadopitysin; (28) rutin; (29) 5′-Butyrylphosphoinosine; (30) evoxine; (31) kaempferol 3-O-β-d-galactoside; (32) lactucin; (33) 1,4-Dimethoxyglucobrassicin; (34) pubesenolide; (35) corchorifatty acid F; (36) linifolin A; (37) N2-(2-Carboxymethyl-2-hydroxysuccinoyl)arginine; (38) 9Z-Octadecenedioic acid; (39) trilobolide; (40) thalidasine; (41) α-linolenic acid; (42) 16-Hydroxy hexadecanoic acid; (43) 4-(3-Hydroxy-7-phenyl-6-heptenyl)-1,2-benzenediol; and (44) (6beta,8betaOH)-6,8-Dihydroxy-7(11)-eremophilen-12,8-olide.

Entry	21	22	23	24	25	26	27	28	29	30	31	32	33	34	35	36	37	38	39	40
**Drug-Likeness**																				
**Lipinski**	Yes	Yes	Yes	Yes	Yes	Yes	Yes	No	Yes	Yes	No	Yes	No	Yes	Yes	Yes	No	Yes	Yes	Yes
**Bioavailability** **score**	0.11	0.56	0.55	0.56	0.55	0.56	0.55	0.17	0.11	0.55	0.17	0.55	0.11	0.55	0.56	0.55	0.11	0.85	0.55	0.55
**Absorption**																				
**Water** **solubility**	−2.338	−0.839	−1.509	−3.683	−2.659	−3.633	−3.02	−2.892	−2.848	−3.23	−2.863	−2.337	−2.811	−5.093	−3.539	−3.27	−2.654	−3.298	−4.682	−4.011
**Caco2** **permeability**	−0.675	−0.386	−0.181	0.879	0.487	0.129	−0.229	−0.949	−0.616	1.195	0.306	0.484	−0.622	0.876	0.747	1.336	−0.53	0.252	0.315	0.468
**Intestinal** **absorption** **(human)**	0.00	25.105	40.251	99.42	95.178	66.637	98.322	23.446	28.681	94.251	48.052	58.69	10.102	94.476	41.903	100.00	0.00	93.188	100.00	94.002
**Skin** **permeability**	−2.735	−2.735	−3.121	−2.889	−3.023	−2.734	−2.735	−2.735	−2.735	−2.806	−2.735	−4.388	−2.735	−3.641	−2.728	−3.337	−2.735	−2.735	−2.833	−2.735
**P-glycoprotein** **substrate**	Yes	No	No	No	No	Yes	Yes	Yes	Yes	Yes	Yes	No	Yes	Yes	No	No	Yes	No	Yes	Yes
**P-glycoprotein I** **inhibitor**	No	No	No	Yes	No	No	Yes	No	No	No	No	No	No	Yes	No	No	No	No	Yes	Yes
**P-glycoprotein II** **inhibitor**	No	No	No	No	No	No	Yes	No	No	No	No	No	No	Yes	No	No	No	No	No	Yes
**Distribution**																				
**VDss** **(human)**	−0.572	−1.009	−0.026	−0.234	−0.056	−0.929	−1.284	−1.663	0.683	−0.15	1.444	−0.098	−0.83	−0.367	−1.174	−0.003	−1.001	−1.452	−0.384	−0.297
**BBB** **permeability**	−1.774	−0.879	−0.611	−0.845	−0.254	−0.424	−1.851	−1.889	−2.225	−0.823	−1.514	−0.169	−1.874	−0.568	−1.032	−0.293	−1.353	−0.441	−0.739	−0.171
**CNS** **permeability**	−3.935	−3.155	−3.205	−3.099	−2.462	−2.326	−3.239	−5.178	−4.043	−3.272	−3.908	−3.031	−4.424	−2.094	−3.545	−2.892	−4.274	−3.007	−3.479	−2.421
**Metabolism**																				
**CYP2D6** **substrate**	No	No	No	No	No	No	No	No	No	No	No	No	No	No	No	No	No	No	No	No
**CYP3A4** **substrate**	No	No	No	Yes	No	Yes	Yes	No	No	No	No	No	No	Yes	No	Yes	No	Yes	No	Yes
**CYP1A2** **inhibitor**	No	No	No	No	No	No	No	No	No	Yes	No	No	No	No	No	No	No	No	No	No
**CYP2C19** **inhibitor**	No	No	No	No	No	No	Yes	No	No	No	No	No	No	No	No	No	No	No	No	No
**CYP2C9** **inhibitor**	No	No	No	No	No	No	Yes	No	No	No	No	No	No	No	No	No	No	No	No	No
**CYP2D6** **inhibitor**	No	No	No	No	No	No	No	No	No	No	No	No	No	No	No	No	No	No	No	No
**CYP3A4** **inhibitor**	No	No	No	No	No	No	No	No	No	No	No	No	No	No	No	No	No	No	No	Yes
**Excretion**																				
**Total** **clearance**	0.394	0.794	0.581	-0.119	0.716	0.76	-0.833	-0.369	0.486	0.638	0.462	0.368	0.507	0.567	2.019	0.417	-0.043	1.835	0.82	0.693
**Renal OCT2** **substrate**	No	No	No	No	No	No	No	No	No	No	No	No	No	No	No	No	No	No	No	No
**Toxicity (Compound Number)**																				
**AMES** **toxicity**	No	No	No	No	No	No	No	No	No	No	No	Yes	No	No	No	Yes	No	No	Yes	No
**Hepatotoxicity**	No	No	No	No	No	Yes	Yes	No	No	Yes	No	No	Yes	Yes	No	No	No	No	No	No
**hERG I** **inhibitors**	No	No	No	No	No	No	No	No	No	No	No	No	No	No	No	No	No	No	No	No
**Skin** **sensitization**	No	No	No	No	No	No	No	No	No	No	No	No	No	No	Yes	No	No	Yes	No	No
**Entry**	**41**	**42**	**43**	**44**																
**Drug-Likeness**																				
**Lipinski**	Yes	Yes	Yes	Yes																
**Bioavailability** **score**	0.85	0.85	0.55	0.55																
**Absorption**																				
**Water** **solubility**	−5.787	−4.518	−3.487	−3.338																
**Caco2** **permeability**	1.577	−1.458	−1.108	0.955																
**Intestinal** **absorption** **(human)**	92.836	91.169	90.306	95.298																
**Skin** **permeability**	−2.722	−2.719	−2.736	−3.799																
**P-glycoprotein** **substrate**	No	No	Yes	No																
**P-glycoprotein I** **inhibitor**	No	No	No	No																
**P-glycoprotein II** **inhibitor**	No	No	Yes	No																
**Distribution**																				
**VDss** **(human)**	−0.617	−0.796	0.635	−0.148																
**BBB** **permeability**	−0.115	−0.36	0.014	−0.315																
**CNS** **permeability**	−1.547	−2.99	−2.236	−2.252																
**Metabolism**																				
**CYP2D6** **substrate**	No	No	No	No																
**CYP3A4** **substrate**	Yes	Yes	Yes	No																
**CYP1A2** **inhibitor**	Yes	No	Yes	No																
**CYP2C19** **inhibitor**	No	No	Yes	No																
**CYP2C9** **inhibitor**	No	No	Yes	No																
**CYP2D6** **inhibitor**	No	No	No	No																
**CYP3A4** **inhibitor**	Yes	No	No	No																
**Excretion**																				
**Total** **clearance**	1.991	1.786	0.136	−1.018																
**Renal OCT2** **substrate**	No	No	No	Yes																
**Toxicity (Compound Number)**																				
**AMES** **toxicity**	No	No	No	Yes																
**Hepatotoxicity**	Yes	No	No	No																
**hERG I** **inhibitors**	No	No	No	No																
**Skin** **sensitization**	Yes	Yes	No	No																

## Data Availability

All data generated or analyzed during this study are included in this article.

## Sample Availability

Samples of the extract is available from the authors.
